# Long-term outcomes and predictors of vedolizumab persistence in ulcerative colitis

**DOI:** 10.1177/17562848241258372

**Published:** 2024-07-30

**Authors:** Beatriz Gros, Hannah Ross, Maureen Nwabueze, Nathan Constantine-Cooke, Lauranne A. A. P. Derikx, Mathew Lyons, Claire O’Hare, Colin Noble, Ian D. Arnott, Gareth-Rhys Jones, Charlie W. Lees, Nikolas Plevris

**Affiliations:** Edinburgh IBD Unit, Western General Hospital, Edinburgh, Scotland, UK; Department of Gastroenterology; Liver and Digestive Diseases Networking Biomedical Research Centre (CIBEREHD), Madrid, Spain and Hepatology, Reina Sofía University Hospital, Córdoba, Spain; Edinburgh IBD Unit, Western General Hospital, Edinburgh, Scotland, UK; Edinburgh IBD Unit, Western General Hospital, Edinburgh, Scotland, UK; MRC Human Genetics Unit, Institute of Genetics and Cancer, University of Edinburgh, Western General Hospital, Edinburgh, Scotland, UK; Centre for Genomics and Experimental Medicine, Institute of Genetics and Cancer, University of Edinburgh, Western General Hospital, Edinburgh, Scotland, UK; Edinburgh IBD Unit, Western General Hospital, Edinburgh, Scotland, UK Inflammatory Bowel Disease Center, Department of Gastroenterology and Hepatology, Radboud University Medical Center, Nijmegen, The Netherlands; Department of Gastroenterology and Hepatology, Erasmus MC, University Medical Centre Rotterdam, Rotterdam, The Netherlands; Edinburgh IBD Unit, Western General Hospital, Edinburgh, Scotland, UK; Edinburgh IBD Unit, Western General Hospital, Edinburgh, Scotland, UK; Edinburgh Pharmacy Unit, Western General Hospital, Edinburgh, UK; Edinburgh IBD Unit, Western General Hospital, Edinburgh, Scotland, UK; Edinburgh IBD Unit, Western General Hospital, Edinburgh, Scotland, UK; Edinburgh IBD Unit, Western General Hospital, Edinburgh, Scotland, UK; Centre for Inflammation Research, The Queen’s Medical Research Institute, University of Edinburgh, Scotland, UK; Edinburgh IBD Unit, Western General Hospital, NHS Lothian, Crewe Road, Edinburgh EH4 2XU, UK; Centre for Genomics and Experimental Medicine, Institute of Genetics and Cancer, University of Edinburgh, Western General Hospital, Edinburgh, Scotland, UK; Edinburgh IBD Unit, Western General Hospital, Edinburgh, Scotland, UK

**Keywords:** real-world evidence, ulcerative colitis, vedolizumab

## Abstract

**Background:**

Long-term vedolizumab (VDZ) outcomes in real-world cohorts have been largely limited to 1-year follow-up, with few bio-naïve patients or objective markers of inflammation assessed.

**Objectives:**

We aimed to assess factors affecting VDZ persistence including clinical, biochemical and faecal biomarker remission at 1, 3 and 5 years.

**Design:**

We performed a retrospective, observational, cohort study.

**Methods:**

All adult inflammatory bowel disease (IBD) patients who had received VDZ induction for ulcerative colitis (UC)/IBD-unclassified (IBDU) were included. Baseline phenotype and follow-up data were collected *via* a review of electronic medical records.

**Results:**

We included 290 patients [UC *n* = 271 (93.4%), IBDU *n* = 19 (6.6%)] with a median time on VDZ of 27.6 months (interquartile range: 14.4–43.2). At the end of follow-up, a total of 157/290 (54.1%) patients remained on VDZ. The median time to discontinuation was 14.1 months (7.0–23.3). Previous exposure to ⩾1 advanced therapy, steroid use at baseline and disease extension (E3 and E2 *versus* E1) were independent predictors for worse VDZ persistence. Clinical remission (partial Mayo < 2) was 75.7% (171/226), 72.4% (157/217) and 70.2% (127/181) at years 1, 3 and 5, respectively. Steroid use during maintenance VDZ therapy occurred in 31.7% (92/290), hospitalization in 15.5% (45/290) and surgery in 3.4% (10/291). The rate of serious adverse events was 1.2 per 100 patient-years of follow-up.

**Conclusion:**

VDZ effectiveness appears enduring with favourable long-term safety profile. VDZ persistence was influenced by previous exposure to biologics/small molecules, disease distribution and steroid use at baseline in our study.

## Plain language summary

Vedolizumab long-term use in ulcerative colitis

What was this study done?

• Vedolizumab efficacy and safety in ulcerative colitis have been firmly established by existing evidence.

• Long-term data from the GEMINI trial further corroborate the favourable safety profile over an extended duration but there is little data on long-term vedolizumab use over 1 year.

What did the researches do?

• We performed a retrospective, observational, cohort study. All adult IBD patients who ever received vedolizumab induction from November 2014 to December 2021 for ulcerative colitis/IBDU were included.

What did the researchers find?

• This real-world study demonstrates that vedolizumab persistence exceeds 80% at 1 year and remains nearly 50% at 5 years with no new safety signals.

• Worse vedolizumab persistence is associated with prior exposure to biologics/small molecules, more extensive disease involvement and steroid use at vedolizumab initiation.

What do the findings mean?

• These findings have important implications for drug positioning and sequencing, as well as for optimizing outcomes when vedolizumab is utilized as first-line therapy. Furthermore, it also emphasizes the long-term safety profile.

## Introduction

Vedolizumab (VDZ), a humanized monoclonal antibody directed against α4β7 integrin, was first approved for the treatment of ulcerative colitis (UC) in 2014.^1^ Its approval came after the phase III GEMINI trial programme, which demonstrated maintenance treatment with VDZ for up to 52 weeks was effective and well tolerated in UC.^2^ However, longer-term data are limited.

Considering the infrequent antibody development against VDZ, it is reasonable to speculate that there might be greater drug persistence and/or better outcomes when compared to anti-tumour necrosis factor (anti-TNF) therapies, which often result in a substantial number of patients discontinuing treatment due to issues related to immunogenicity, primary/secondary nonresponse and intolerance.^3^ Indeed, the VARSITY controlled trial comparing the efficacy of VDZ and adalimumab in UC suggested superior outcomes in VDZ-treated patients at 52 weeks.^4^

Long-term outcomes with VDZ to date are limited to controlled trials that primarily assessed safety and real-world data limited to 2 years of follow-up with predominantly bio-experienced patients.^5–7^ Furthermore, the factors associated with drug persistence are poorly understood and are not always shared between UC and Crohn’s disease.^8^ As such, the long-term outcomes of VDZ treatment in a mixed biologic naïve/experienced population remain unclear.

In the Edinburgh IBD Unit, VDZ has been used for over 8 years in UC, with a significant number of UC patients receiving it as a first-line advanced treatment. This study aimed to assess the long-term effectiveness, predictive factors associated with drug persistence and the safety of long-term VDZ in patients with UC.

## Methods

### Study design

This was a retrospective, observational, cohort study performed at the Edinburgh IBD Unit, a tertiary inflammatory bowel disease (IBD) referral centre within NHS Lothian (Scotland). NHS Lothian provides universal, free point-of-care healthcare for a population of 916,310 people (2021), including a rigorously validated prevalent population of 10,499 patients with IBD in 2019.^9^

### Participants

We identified all adult (>18 years old) IBD patients receiving VDZ from November 2014 to December 2021 *via* pharmacy and electronic medical health records (TrakCare patient management ©InterSystems) with follow-up until January 2023. Inclusion criteria were (i) a confirmed diagnosis of UC (based on standard criteria) or IBD-unclassified (IBDU) favouring UC [pathology reported as consistent with UC^10^ but with either perianal disease or atypical distribution for UC (i.e. rectal sparing)]; (ii) completion of standard induction dosing (0, 2 and 6 weeks) and at least one clinical assessment post-induction. Patients with Crohn’s disease, IBD-U favouring Crohn’s disease, microscopic colitis or receiving VDZ treatment for pouchitis were excluded. There were five patients who did not complete VDZ induction and were also excluded from our analysis.

### Data collection

Baseline phenotyping and follow-up data were collected *via* review of electronic medical records. Data regarding clinical disease activity scores (partial Mayo score), C-reactive protein (CRP), faecal calprotectin (FC), dose adjustments, steroid prescriptions, hospitalization rates, surgical rates and adverse events were collected. All FC samples were measured at the Western General Hospital, Edinburgh using a standard enzyme-linked immunosorbent assay (Calpro AS, Lysaker, Norway).

### Primary and secondary outcomes

The primary outcome of this study was VDZ persistence. Secondary outcomes included the following: clinical remission, biochemical remission and faecal biomarker remission at year 1 (±8 weeks), year 3 (±8 weeks), year 5 (±8 weeks) and last follow-up; change in partial Mayo, CRP and FC during follow-up; hospitalization rates; surgical rates; baseline predictors of VDZ persistence and serious adverse events.

### Definitions

Clinical remission, biochemical remission and faecal biomarker remission were defined as a partial Mayo ⩽ 1, CRP ⩽ 5 mg/L and FC < 250 µg/g, respectively. Primary non-response (PNR) was defined as a lack of clinical and biochemical response from VDZ initiation resulting in treatment discontinuation in those receiving full VDZ induction. Secondary loss of response was defined as clinical and biochemical relapse in patients who previously responded as confirmed by normalization of biochemical (CRP ⩽ 5 mg/L/FC < 250 µg/g) and/or clinical parameters (partial Mayo ⩽ 1) leading to drug discontinuation. Patients who were initially on steroids and did not appropriately taper their dosage (with an initial steroid course lasting approximately 8 weeks) or were prescribed an increased steroid dosage during the initial course were categorized as receiving a new steroid prescription whenever this adjustment took place. Comorbidity data were collected pertaining to active cancer, history of cancer, heart failure (New York Heart Association > III–IV), demyelinating disease or family history of demyelinating disease, high risk of tuberculosis (defined as positive quantiFERON and/or recent contact with individuals with known active tuberculosis) and advanced liver disease (either compensated or decompensated liver cirrhosis). Steroid prescription during follow-up was defined as oral prednisone or budesonide prescribed at any point after VDZ induction, topical steroids were not considered. VDZ intensification was not based on TDM but based on active clinical symptoms and increase in CRP and/or FC. Serious adverse events were defined as any event leading to disruption or discontinuation of therapy, hospitalization or death.

### Statistical analysis

SPSS Version 25 (IBM Inc., Armonk, NY, USA) and Prism Version 7.0 (Graphpad Software, San Diego, CA, USA) were used for statistical analyses and the generation of graphs. Descriptive statistics are presented as medians with interquartile range (IQR) for continuous variables, and frequencies with percentages for categorical variables. For comparison of non-parametric continuous variables, the Mann–Whitney *U* or Kruskal–Wallis test was used where appropriate and the Wilcoxon test for paired data. For the comparison of categorical variables, the Chi-squared test was used. Survival analysis was performed using Kaplan–Meier analysis, and comparisons were made using the log-rank test. Patients were censored at discontinuation or the last follow-up. For the effectiveness outcomes, analysis was performed on patients with available data. An intention-to-treat analysis with non-responder imputation (NRI) was also performed. Cox proportional hazard regression analyses were carried out to identify possible baseline predictors of drug survival. Variables for analysis were chosen *a priori* (Supplemental Table 1). In the case of a *p* value of <0.1 in univariable analysis, variables were included in the multivariable analysis. A *p* value of <0.05 was considered statistically significant.

Effective outcomes are presented as observed with available data, further analysis was performed using both the last observation carried forward and NRI which gives a more conservative estimate of the effectiveness up to the 3 years of follow-up.

Data were reported according to Strengthening the Reporting of Observational Studies in Epidemiology guidelines.^11^

## Results

### Study population

A total of 290 patients [UC *n* = 271 (93.4%), IBDU *n* = 19 (6.6%)] were identified, with a median duration of 27.6 months (IQR 14.4–43.2) on VDZ (Table 1). A total of 171 patients (59%) were male. The median disease duration was 10 years (IQR 6–17 years) and the median age at VDZ initiation was 49.6 years (IQR 35.2–64.4 years) including 71 patients (21.4%) > 65 years old. Most patients had extensive (E3) disease [*n* = 157 (55.1%)]. Of the 290 patients, 184 (63.7%) were biologic and small molecule naïve.

**Table 1.** Baseline characteristics of patients on vedolizumab.

Variable	Total cohort (*N* = 290)	Remain VDZ (*n* = 157)	VDZ withdrawal (*n* = 133)	*p* Value
Sex, male, *n* (%)	171 (59)	88 (56.1)	83 (62.4)	0.27
Age at VDZ start, median (IQR)	49.6 (35.2–64.4)	51.3 (34.9–65.7)	48.5 (35.4–62.4)	0.79
Age over 65, *n* (%)	71 (24.5)	44 (28)	27 (20.3)	0.13
BMI, kg/m^2^, median (IQR)^a^	27 (24.1–30.8)	27.1 (24.2–31.1)	26.8 (24.0–30.3)	0.67
Disease duration, years, median (IQR)	10 (6–17)	11 (6–22)	9 (6–14)	0.061
Disease type				0.41
Ulcerative colitis, *n* (%)	271 (93.4)	145 (92.4)	126 (94.7)	
IBD-U, *n* (%)	19 (6.6)	12 (7.6)	7 (5.3)	
Ulcerative colitis Montreal classification, *n* (%)				0.024
E1	23 (8.1)	18 (11.8)	5 (3.8)	
E2	105 (36.8)	58 (38.2)	47 (35.3)	
E3	157 (55.1)	76 (50)	81 (60.9)	
Extraintestinal manifestation, *n* (%)	87 (30.3)	45 (28.7)	42 (14.5)	0.31
Joints	41 (14.1)	26 (16.6)	15 (11.4)	
Skin	4 (1.4)	1 (0.6)	3 (2.3)	
Mouth ulcers	6 (2.1)	4 (2.5)	2 (1.5)	
PSC	13 (4.5)	6 (3.8)	7 (5.3)	
Eye	6 (2.1)	3 (1.9)	3 (2.3)	
Other/more than 1	17 (5.8)	5 (3.2)	12 (9.1)	
Smoking habit, *n* (%)^b^				0.44
Never smoker	133 (45.9)	71 (45.2)	62 (46.6)	
Former smoker	44 (15.2)	19 (12.1)	25 (18.8)	
Active smoker	16 (5.5)	7 (4.5)	9 (6.8)	
Comorbidities of interest, *n* (%)				0.38
Active cancer	13 (4.5)	7 (4.5)	6 (4.5)	
Previous cancer	11 (3.8)	6 (3.8)	5 (3.8)	
Heart failure	8 (2.8)	4 (2.5)	4 (3)	
Neurological disease	2 (0.7)	1 (0.6)	1 (0.8)	
High risk of tuberculosis	8 (2.8)	5 (3.2)	3 (2.3)	
Advanced liver disease	7 (2.4)	1 (0.6)	6 (4.5)	
Previous biologic/small molecules, *n* (%)^c^				
None	184 (63.7)	110 (70.5)	74 (55.6)	0.009
Anti-TNF (infliximab, golimumab, adalimumab)	97 (33.5)	43 (27.4)	54 (40.6)	0.019
Tofacitinib	14 (4.8)	6 (3.8)	8 (6)	0.39
Ustekinumab	4 (1.4)	0	4 (3.2)	0.029
Number of previous biologic/small molecules, *n* (%)^c^				
None	184 (63.7)	110 (70.5)	74 (55.6)	0.005
One	81 (28)	38 (24.2)	43 (32.3)	0.13
Two or more	24 (8.3)	8 (5.1)	16 (12)	0.034
Concomitant immunomodulation at VDZ prescription, *n* (%)^d^				0.43
None	224 (78.6)	123 (80.4)	101 (75.9)	
Thiopurines (AZA, Mercaptopurine)	57 (20)	28 (18.3)	29 (22)	
Methotrexate	4 (1.4)	2 (1.4)	2 (1.5)	
Concomitant steroids at VDZ prescription, *n* (%)^e^	173 (60.7)	83 (52.9)	91 (68.9)	0.015
Baseline partial Mayo ⩾2, *n* (%)^e^	226 (79.6)	113 (72)	113 (85)	0.019
Baseline CRP >5 mg/L, *n* (%)^f^	119 (41.6%)	65 (41.4)	54 (40.9)	0.78
Baseline FC ⩾ 250 µg/g, *n* (%)^g^	201 (84.5%)	99 (83.2)	102 (85.7)	0.59
Baseline albumin <36 g/L, *n* (%)^h^	85 (31.3%)	44 (29.9)	41 (30.8)	0.64

a Missing data *n* = 45.

b Missing data *n* = 96.

c Missing data *n* = 1.

d Missing data *n* = 5.

e Missing data *n* = 6.

f Missing data *n* = 3.

g Missing data *n* = 52.

h Missing data *n* = 17.

Anti-TNF, anti-tumour necrosis factor; AZA, Azathioprine; BMI, body mass index; CRP, C-reactive protein; FC, faecal calprotectin; PSC, Primary Sclerosing Cholangitis; IBDU, inflammatory bowel disease-unclassified; IQR, interquartile range; VDZ, vedolizumab.

### VDZ persistence

**Figure 1.**
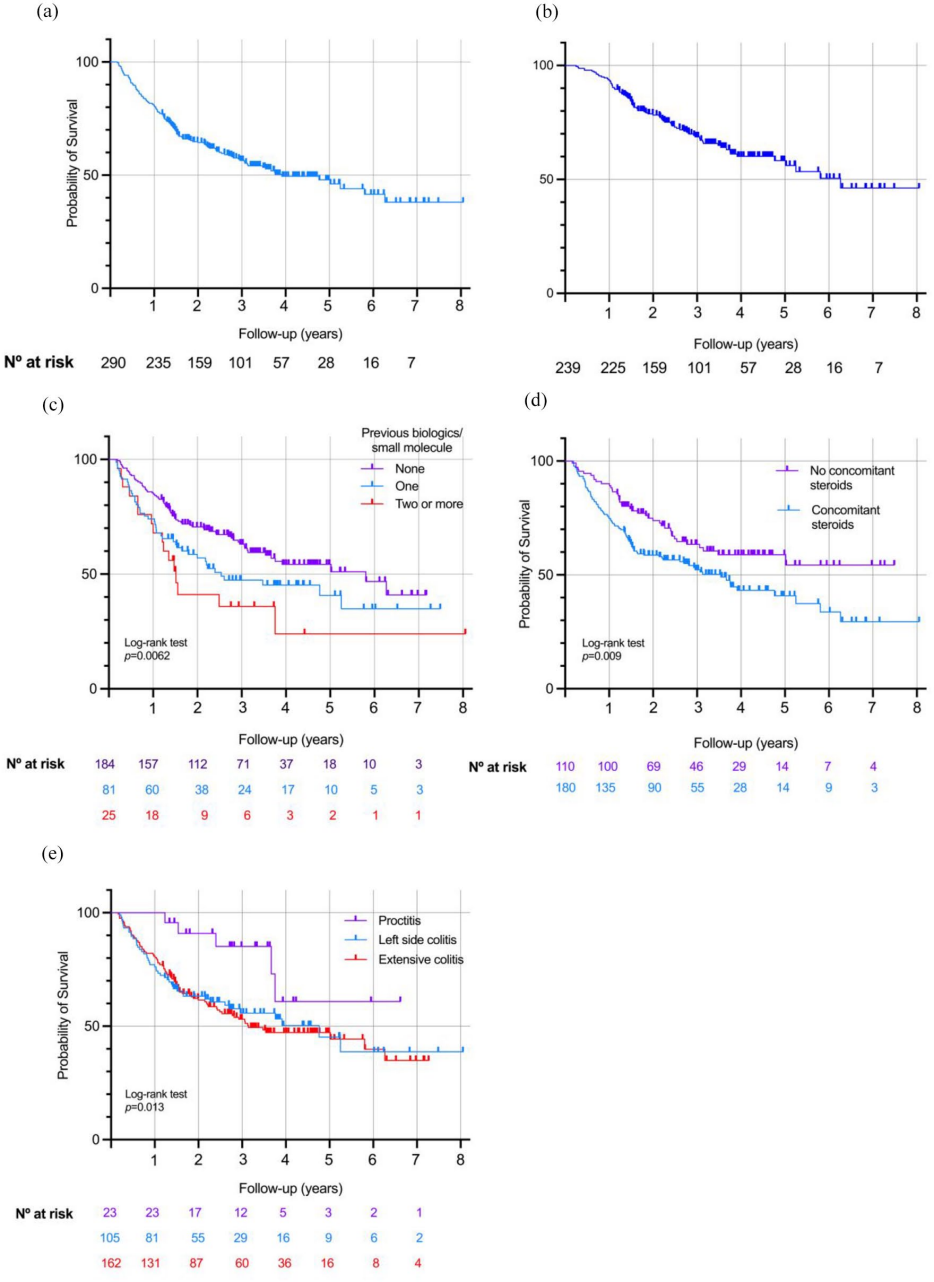
(a) Kaplan–Meier curve for vedolizumab persistence across the total cohort. (b) Kaplan–Meier curve for vedolizumab persistence excluding primary non-response. (c) Kaplan–Meier curve stratified by a number of previous biologic/small molecules. (d) Kaplan–Meier curve stratified depending on the need for steroids at baseline. (e) Kaplan–Meier curve stratified depending on disease extension.

Median time on VDZ of 27.6 months (IQR 14.4–43.2). At the end of follow-up, a total of *n* = 157/290 (54.1%) patients remained on VDZ. VDZ persistence at 1 year was 80.7% (234/290), 64.4% (187/290) at 2 years, 56.5% (164/290) at 3 years, 49.5% (144/290) at 4 years, 47.9% (139/290) at 5 years and 41.5% (120/290) at 6 years (Figure 1). The median time to VDZ discontinuation was 14.1 months (IQR 7.0–23.3 months) (Figure 1). Reasons for drug discontinuation included the following: PNR: 17.6% (51), secondary loss of response: 21.7% (63), adverse events: 3.1% (9), long-term remission: 1.4% (4) and other reasons: 2.1% (6) (patient’s decision *n* = 2, need of drug change to treat another autoimmune disorder *n* = 4).

Secondary loss of response occurred in 2.8% (8/290) patients during the first year of therapy; 11.4% (33/290) patients between the first and second year; 4.5% (13/290) patients between the second and third year; 1.7% (5/290) patients between the third and fourth year and 1.4% (4/290) over the fourth year (Figure 1). When excluding PNR, the median time to VDZ discontinuation was 18.6 months (12.8–32.1).

### Predictors for drug persistence

Univariable Cox regression analysis identified the following factors associated with lower VDZ drug persistence; one previous biologic/small molecule [hazard ratio (HR): 1.53, 95% confidence interval (CI): 1.05–2.22, *p* = 0.028]; two or more previous biologics/small molecules (HR: 2.26, 95% CI: 1.31–3.88, *p* = 0.003), concomitant steroids at baseline (HR: 1.67, 95% CI: 1.16–2.42, *p* = 0.006); partial Mayo score ⩾ 2 at baseline (HR: 1.81, 95% CI: 1.11–2.94, *p* = 0.017); left-side (E2) colitis (HR: 2.72, 95% CI: 1.08–6.83, *p* = 0.034) and extensive (E3) colitis (HR: 2.94, 95% CI: 1.19–7.23, *p* = 0.019). Multivariable Cox regression analysis identified previous exposure to one biologic/small molecule (HR: 1.54, 95% CI: 1.05–2.25, *p* = 0.028), previous exposure to two or more biologic/small molecule (HR: 2.12, 95% CI: 1.25–3.71, *p* = 0.006), concomitant steroids at baseline (HR: 1.54, 95% CI: 1.05–2.27, *p* = 0.027), left-side (E2) colitis (HR: 2.82, 95% CI: 1.12–7.13, *p* = 0.028) and extensive (E3) colitis (HR: 3.40, 95% CI: 1.37–8.42, *p* = 0.008) as independent predictors for VDZ persistence (Table 2 and Figure 1).

**Table 2.** Variables associated with vedolizumab withdrawal.

Variable	Univariable Cox regression			Multivariable Cox regression		
	Hazard ratio	95% CI	*p*	Hazard ratio	95% CI	*p*
Disease extension						
E1: distal (reference)			0.022			0.025
E2: left side	2.72	1.08–6.83	0.034	2.82	1.12–7.13	0.028
E3: extensive	2.94	1.19–7.23	0.019	3.40	1.37–8.42	0.008
Number of previous biologic/small molecule						
None (reference)			0.004			0.007
One	1.53	1.05–2.22	0.028	1.54	1.05–2.25	0.028
Two or more	2.26	1.31–3.88	0.003	2.12	1.25–3.71	0.006
Concomitant steroids at baseline	1.67	1.16–2.42	0.006	1.54	1.05–2.27	0.027
Partial Mayo ⩾ 2 at baseline	1.81	1.11–2.94	0.017			

CI, confidence interval.

Of 133 patients who withdrew VDZ, 69.2% (92) were switched to a different drug (43 tofacitinib, 20 ustekinumab, 19 infliximab, 6 filgotinib and 4 adalimumab); 12 (9%) had surgery (*n* = 10 urgent, *n* = 2 elective); 22 (16.5%) did not receive additional advanced therapies (4 were in remission, 6 were stopped due to adverse events, 2 discontinued treatment at their own volition, 1 liver transplant, 1 stopped VDZ and started rituximab for another condition, 4 lost response and received steroid rescue and decided to continue on mesalazine after the steroids, 4 lost response and did not start any other therapy for unknown reasons); 7 (5.3%) patients were lost to follow-up or were transferred to other centres where data were not available.

### Effectiveness outcomes

**Figure 2.**
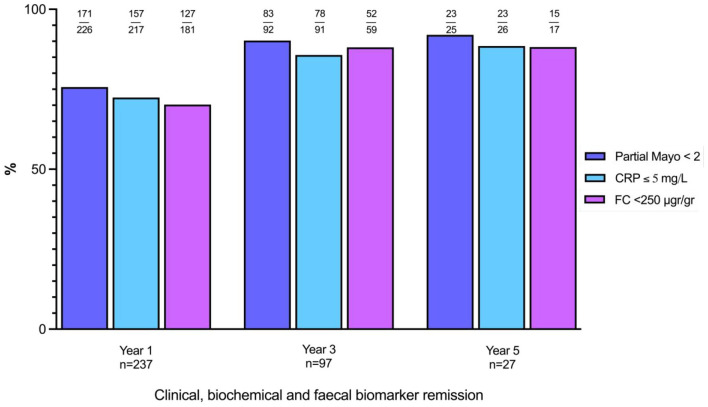
Clinical, biochemical and FC biomarker remission at year 1, year 3 and year 5 from vedolizumab initiation. CRP, C-reactive protein; FC, faecal calprotectin.

**Figure 3.**
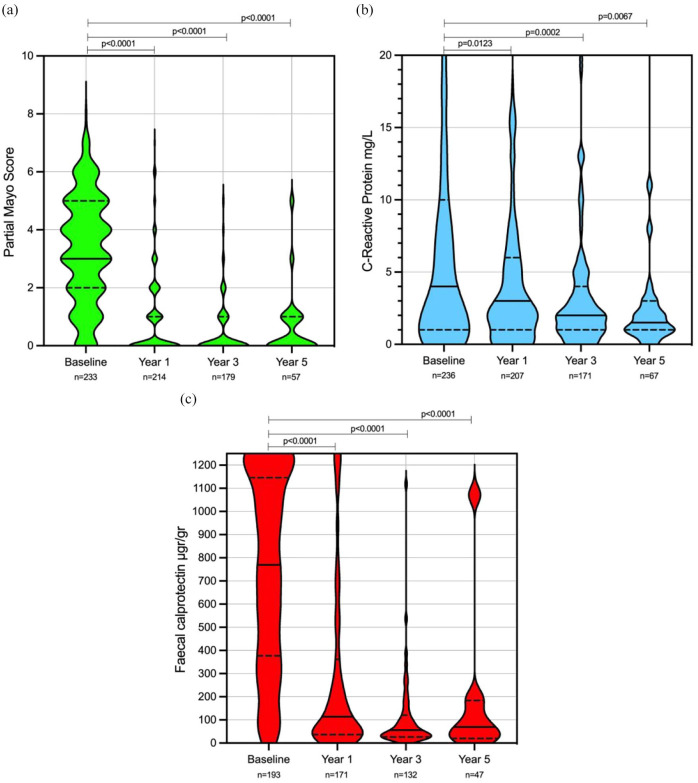
CRP, faecal calprotectin and partial Mayo score, at baseline, year 1, year 3, year 5 from vedolizumab prescription. Violin plots show median (solid line), IQR (dotted line), maximum and minimum. CRP outliers were removed for graph representation but accounted for statistical comparison: at baseline, there were 34 outliers; 10 at year 1; none at year 3; 1 at year 5. Mann–Whitney *U* test was used to compare each baseline median compared to years 1, 3 and 5. CRP, C-reactive protein; FC, faecal calprotectin.

Of the 290 patients, 238 (82.1%) were on VDZ at 1 year. Clinical remission, biochemical remission and faecal biomarker remission rates were 75.7% (171/226), 72.4% (157/217) and 70.2% (127/181) at 1 year, respectively (Figure 2). A total of 97 patients (33.4%) were on VDZ at 3 years. Clinical remission, biochemical remission and faecal biomarker remission rates were 90.2% (83/92), 85.7% (78/91) and 88.1%(52/59) at year 3, respectively (Figure 2). Finally, 27 patients (9.3%) were on VDZ for at least 5 years. Clinical remission, biochemical remission and faecal biomarker remission were 92% (23/25), 88.5% (23/26) and 88.2%(15/17) at year 5, respectively (Figure 2). At last follow-up clinical remission, biochemical remission and faecal biomarker remission rates were 57.8% (167/290), 68.6% (199/290) and 54.3% (152/290), respectively. There was a significant reduction in median partial Mayo, CRP and FC at 1, 3 and 5 years and last follow-up when compared to baseline (Supplemental Table 2 and Figure 3). Utilizing NRI analysis, conservative effectiveness rates were also calculated (Supplemental Table 2 and Figure 2). Clinical, biochemical and faecal biomarker remission at year 1 and year 3 were 59% (171), 54.1% (157), 43.8% (127) and 28.6% (83), 26.9% (78), 17.9% (52), respectively.

### Steroid prescription, dose intensification, hospitalization and surgery

During follow-up, 92 (31.7%) patients required steroid prescription. The median time from VDZ initiation to the first steroid course for these patients was 9.6 months (5.1–21.6). Of those who required steroids during follow-up, 75 (76.5%) withdrew VDZ, whereas 23 (23.5%) remained on it by the end of follow-up.

Among those who ever received a steroid course during follow-up, 33.7% (33/98) was due to PNR and 66.3% (65/98) was due to secondary loss of response. Among those with secondary loss of response, 38.5% (24/65) remained on VDZ at the end of follow-up.

A total of 56 (19.3%) patients needed VDZ intensification to every 4 or 6 weeks, with a median time to drug intensification of 10.2 months (7.1–16.4). Of these patients, 42 (75%) discontinued VDZ during follow-up and 14 (25%) remained on it at the end of the follow-up.

Among those who ever needed VDZ intensification, 21.4% (12/56) was due to PNR and 78.6% (44/56) was due to secondary loss of response. Of those with secondary loss of response receiving dose intensification, 38.6% (17/44) remained on VDZ at the end of follow-up.

Finally, 45 (15.5%) patients needed hospital admission of whom 10 (3.5%) patients needed a total colectomy. The median time from VDZ initiation to first hospitalization was 8.8 months (5.3–18.1) and to surgery 9.5 months (5.1–16.1). Among those needing hospital admission, 35 (77.8%) withdrawn the drug whereas 10 (3.4%) remained on the drug until the end of follow-up (Supplemental Figure 3).

### Safety

Adverse events were documented in 35 patients (4.8 per 100 patient-year follow-up). The majority of adverse events were mild [skin reactions (rash, pruritus, erythema after subcutaneous injection), arthralgia and upper respiratory infection] (Table 3). Serious adverse events leading to drug withdrawal were documented in nine patients (1.2 per 100 patient-years of follow-up) (Table 3). No new safety signals were identified.

**Table 3.** Adverse events during follow-up.

Type of adverse event	*n* (%)
Any adverse event	35 (12.1)
Skin reaction	12 (4.1)
Respiratory tract infection	8 (2.8)
Arthralgia	6 (2.1)
COVID-19 infection	3 (1.0)
Hair loss	2 (0.7)
Nasopharyngitis	1 (0.4)
Headache	1 (0.4)
Acute diverticulitis treated conservatively	1 (0.4)
Campylobacter gastroenteritis	1 (0.4)
*Salmonella* gastroenteritis	1 (0.4)
Persistent cough	1 (0.4)
Adverse events leading to drug withdrawal *n* = 9	
Respiratory tract infection needing admission	2 (0.7)
Arthralgia	1 (0.4)
Skin reaction	3 (1.0)
Skin reaction and headache	1 (0.4)
Skin reaction and arthralgia	1 (0.4)
Persistent cough	1 (0.4)

## Discussion

In this study, we present results from a large, real-world cohort investigating the long-term persistence, effectiveness and safety of VDZ for the treatment of patients with UC/IBDU. To our knowledge, our real-world cohort has the longest follow-up and greatest proportion of biologic/small molecule naïve patients to date. Our data show that VDZ persistence was >80% at 1 year and remained nearly 50% at 5 years. Exposure to one or more prior biologics, extensive disease (E2 and E3) and use of steroids at baseline increased the risk for VDZ discontinuation. In addition, 19% of patients needed treatment intensification, and 31% needed steroids during follow-up, of whom 75% and 76.5%, respectively, ultimately failed therapy. With 4.8 per 100 patient-year follow-ups, no new safety signals were observed. These data add to the growing body of real-world evidence of VDZ and help better our understanding of the longer-term outcomes in UC.

Data on long-term outcomes with VDZ therapy are lacking. Current studies have focused on 52-week follow-ups with limited data on objective markers of inflammation.^6^ The seminal GEMINI trials in UC showed a clinical response at induction of 47.1% and clinical remission at 52 weeks of 44.8%.^2,5^ Thereafter, the GEMINI long-term safety trial, which was a single-arm, continuing phase III trial investigating the safety of VDZ in IBD, reported data for up to 8 years. In UC, 35% of patients had a clinical response, while 33% of patients were in clinical remission at week 400. However, this study was not specifically designed or powered to evaluate efficacy outcomes. In addition, RCTs do not always reflect the patient population seen in clinical practice as, for example, patients with proctitis alone are generally not included.^2,12^ This is of importance as most of the evidence from advanced therapies for proctitis come from real-world experiences.^13^ Many real-world studies investigating VDZ, including ours, typically involve a limited number of patients with proctitis receiving the drug.^14–16^ This limitation hampers our comprehension of its effectiveness in this particular context. In our study, only 8% of the cohort had proctitis, and its presence was linked to a more sustained response. Nonetheless, it is imperative to approach these findings with caution, as these patients likely also received topical mesalazine and could have a less aggressive disease compared to more extensive forms, which could potentially influence outcomes. Unfortunately, we did not gather specific data on topical therapy, thus leaving a gap in our understanding of ulcerative proctitis optimal management.

One of the most extensive real-world data reported to date comes from a prospective study from the French IBD group GETAID.^17^ This study encompassed a cohort of IBD patients of which more than 97% had prior exposure to anti-TNF therapy. The study reported VDZ persistence rate in UC of 42.9% at 3 years. In our study, we observed a markedly higher persistence rate at 3-year with a recorded value of 56.5%. This difference can be attributed to the substantial proportion of biologic-naïve patients within our cohort, who are typically more likely to respond as compared to biologic-experienced patients.^2^

Furthermore, a recent meta-analysis of observational real-world data studies, focused on VDZ treatment in UC, presented pooled estimate rates for clinical remission of 40% at induction and 45% at year-1.^16^ In our cohort, at the end of follow-up [27.6 months (IQR 14.4–43.2)], we observed notably elevated rates of clinical remission, biochemical remission and faecal biomarker remission at 57.8% (167/290), 68.6% (199/290) and 54.3% (152/290), respectively. These rates are comparably higher, as previously mentioned these differences may be attributable to the large number of biologic-naïve within our cohort.

Sequencing of UC therapies has become a topic of great debate.^18^ In our study, we demonstrated that the greater the number of prior biologics an individual was exposed to the higher the risk of treatment failure (one prior biologic/small molecule: HR 1.54; two or more biologic/small molecule: HR 2.12). These findings, that earlier VDZ use in drug sequencing may be beneficial, have been demonstrated in several other cohorts including our previously reported pan-Scottish real-world experience.^19–21^ The VARSITY trial has also demonstrated the VDZ was superior to adalimumab in achieving clinical remission and endoscopic improvement in moderate–severe UC.^4^ Furthermore, in the real-world EVOLVE study, they also demonstrated that unadjusted rates of treatment persistence, clinical response and clinical remission at 6 months were similar in patients receiving anti-TNF first line *versus* those receiving anti-TNF following VDZ failure.^22^ Thus, an argument can be made for the use of VDZ as the first line in UC.

In our multi-variable analysis, we also demonstrated that steroids at baseline (HR: 1.57, 95% CI: 1.08–2.29, *p* = 0.019), and more extensive disease [E2 (HR: 2.80, 95% CI: 1.11–7.09, *p* = 0.029) and E3 disease (2.37, 95% CI: 1.37–4.09)] were independently associated with drug persistence. Regarding steroid use at baseline, this may probably be a reflection of patients who have severe disease, interestingly we did not find an association to CRP, partial Mayo or FC values at baseline.

With anti-TNF treatment, immunogenicity is one of the most common causes of secondary loss of response; however, studies have shown very low immunogenicity rates with VDZ.^23^ It is unclear why secondary loss of response occurs in certain patients on VDZ but this could just be a reflection of the natural progression of the disease. Finally, we did not find any association between baseline immunosuppressant use and VDZ persistence. However, it is imperative to approach this finding cautiously, given our clinical practice of discontinuing immunosuppressants agents upon the establishment of VDZ therapy. Recent research, such as a prospective study in patients with Crohn’s disease^24^ and preliminary data from a randomized controlled trial in patients with UC,^25^ hints at a potential advantage in maintaining combination therapy in certain cases. To shed further light on this matter, additional studies on this specific scenario are needed.

One of the favourable characteristics of VDZ is gut selectivity and a subsequently favourable safety profile. As such, in many centres, it is favoured over anti-TNF when used in the elderly. However, this population is often not represented in clinical trials with many trials having upper age limits.^26^ In our study, we showed age was not related to drug persistence. This is aligned with Yajnik *et al*.^27^’s *post hoc* analysis of the GEMINI trials that showed no differences in VDZ safety and efficacy when stratifying by age. In addition, Cohen *et al.*^28^ also showed that VDZ was equally effective in both young and older patients with IBD.

Regarding safety, a recent meta-analysis reported a pooled incidence of adverse events of 34.6 per 100 person-years (range: 3.5–274.8; *I*^2^ = 96%), which is higher compared to our study.^16^ These differences are probably explained by mild reactions that might not being documented; this is one of the known limitations of the retrospective nature of our study. However, no new safety signals were identified compared to previously reported studies.^21,29,30^

Our study has several strengths including its large sample size, the long follow-up and the large number of biologic/small molecule naïve patients. Nonetheless, there are some limitations to our study. Firstly, this is a retrospective study performed at a single centre. There were also incomplete data for effectiveness outcomes with varying follow-up due to the retrospective nature. However, we did perform NRI analysis to try and present conservative estimates of effectiveness. Endoscopic data assessment was not collected as the defined intervals for follow-up (±8 weeks at years 1, 3 and 5) provided not enough data for analysis.

## Conclusion

VDZ is safe and effective in the long-term with better persistence observed in biological/small molecule naïve patients, those with shorter disease extension and those who did not need steroids at induction.

## Supplemental Material

sj-docx-1-tag-10.1177_17562848241258372 – Supplemental material for Long-term outcomes and predictors of vedolizumab persistence in ulcerative colitis Supplemental material, sj-docx-1-tag-10.1177_17562848241258372 for Long-term outcomes and predictors of vedolizumab persistence in ulcerative colitis by Beatriz Gros, Hannah Ross, Maureen Nwabueze, Nathan Constantine-Cooke, Lauranne A. A. P. Derikx, Mathew Lyons, Claire O’Hare, Colin Noble, Ian D. Arnott, Gareth-Rhys Jones, Charlie W. Lees and Nikolas Plevris in Therapeutic Advances in Gastroenterology

## Declarations

**Ethics approval and consent to participate:** This work was considered a service evaluation/audit as all data were collected as part of routine clinical care. Therefore, no written consent or formal ethical approval was necessary as per departmental policy and Health Research Authority.^31^ This study was done according to the Declaration of Helsinki.

**Consent for publication:** Not applicable.

**Availability of data and materials:** All data are incorporated into the article and its online Supplemental Material.
