# Supplementary material for: Long-term outcomes and predictors of vedolizumab persistence in ulcerative colitis
Source: Ther Adv Gastroenterol. 2024 Jul 30;17:17562848241258372. doi: 10.1177/17562848241258372 (PMC11289824; doi:10.1177/17562848241258372)
Supplement: sj-docx-1-tag-10.1177_17562848241258372 – Supplemental material for Long-term outcomes and predictors of vedolizumab persistence in ulcerative colitis [file sj-docx-1-tag-10.1177_17562848241258372.docx]

**Supplementary material**

**TABLE 1.**

| **Variables evaluated in the univariable analysis** |
| --- |
| Gender |
| Age at diagnosis |
| Type of disease (UC/IBDU) |
| Smoking habit |
| Body Mass Index |
| Age over 65 years old |
| Concomitant immunosuppressant therapy |
| Extra intestinal manifestations |
| C-Reactive Protein at baseline |
| Faecal calprotectin at baseline |
| Albumin level at baseline |
| Disease extension |
| Number of biologics/small molecules |
| Concomitant steroids at baseline |
| Partial Mayo score at baseline |

**TABLE 2**

| **Variable** | Baseline | Year 1  LOCF | Year 1  NRI | Year 3  LOCF | Year 3  NRI | At last clinical observation |  |
| --- | --- | --- | --- | --- | --- | --- | --- |
| **Clinical disease activity** | | | | | | | |
| Partial Mayo score, median (IQR) | 3 (2-5) | 1 (0-2.25) | 0 (0-1) | 1 (0-3) | 0 (0-1) | 1 (0-3) |  |
| pMayo <2, n (%) | 58 (20) | 176 (61.5) | 171 (59) | 173 (59.9) | 83 (28.6) | 167 (57.8) |  |
| **Biochemical and faecal biomarker disease activity** | | | | | | | |
| C-Reactive protein, mg/L, median (IQR) | 4 (1-10) | 3 (1-7) | 3 (1-6) | 3 (1-8) | 2 (1-4) | 3 (1-9) |  |
| CRP ≤5 mg/L. n (%) | 168 (57.9) | 197 (67.9) | 157 (54.1) | 201 (69.3) | 78 (26.9) | 199 (68.6) |  |
| FC µgr/gr, median /IQR) | 770 (377-1146) | 210 (50-780) | 114 (37-362) | 210 (42-929) | 56 (26-128) | 199 (42-932) |  |
| FC <250 µgr/gr, n (%) | 37 (12.8) | 144 (54.1) | 127 (43.8) | 147 (53.8) | 52 (17.9) | 152 (54.3) |  |

Clinical, biochemical and faecal calprotectin remission at baseline, year 1 and year 3. LOCF (last observation carried forward); NRI (non-responder imputation)

**TABLE 3**

| **Variable** | **Univariable Cox Regression** | | | **Multivariable Cox Regression** | | |
| --- | --- | --- | --- | --- | --- | --- |
|  | Hazard Ratio | 95% CI | *p* | Hazard Ratio | 95% CI | *p* |
| Disease extension  - E1-E2 (reference)  - E3: extensive | 1.09 | 0.63-1.90 | 0.76 |  | | |
| Number of previous biologic/small molecule  - None (reference)  - One  - Two or more | 2.29  3.55 | 1.25-4.19  1.63-7.70 | 0.002  0.007  0.001 | 3.02  3.40 | 1.52-5.98  1.42-8.14 | 0.002  0.002  0.006 |
| Concomitant steroids at baseline | 4.25 | 1.91-9.45 | 0.0001 | 2.97 | 1.27-6.95 | 0.012 |
| Partial Mayo ≥ 2 at baseline | 14.38 | 1.98-104.08 | 0.008 | 9.48 | 1.12-72.38 | 0.030 |

Univariable and multivariable Cox Regression analysis of the variables associated to primary non-response.

**FIGURE 1.**

**
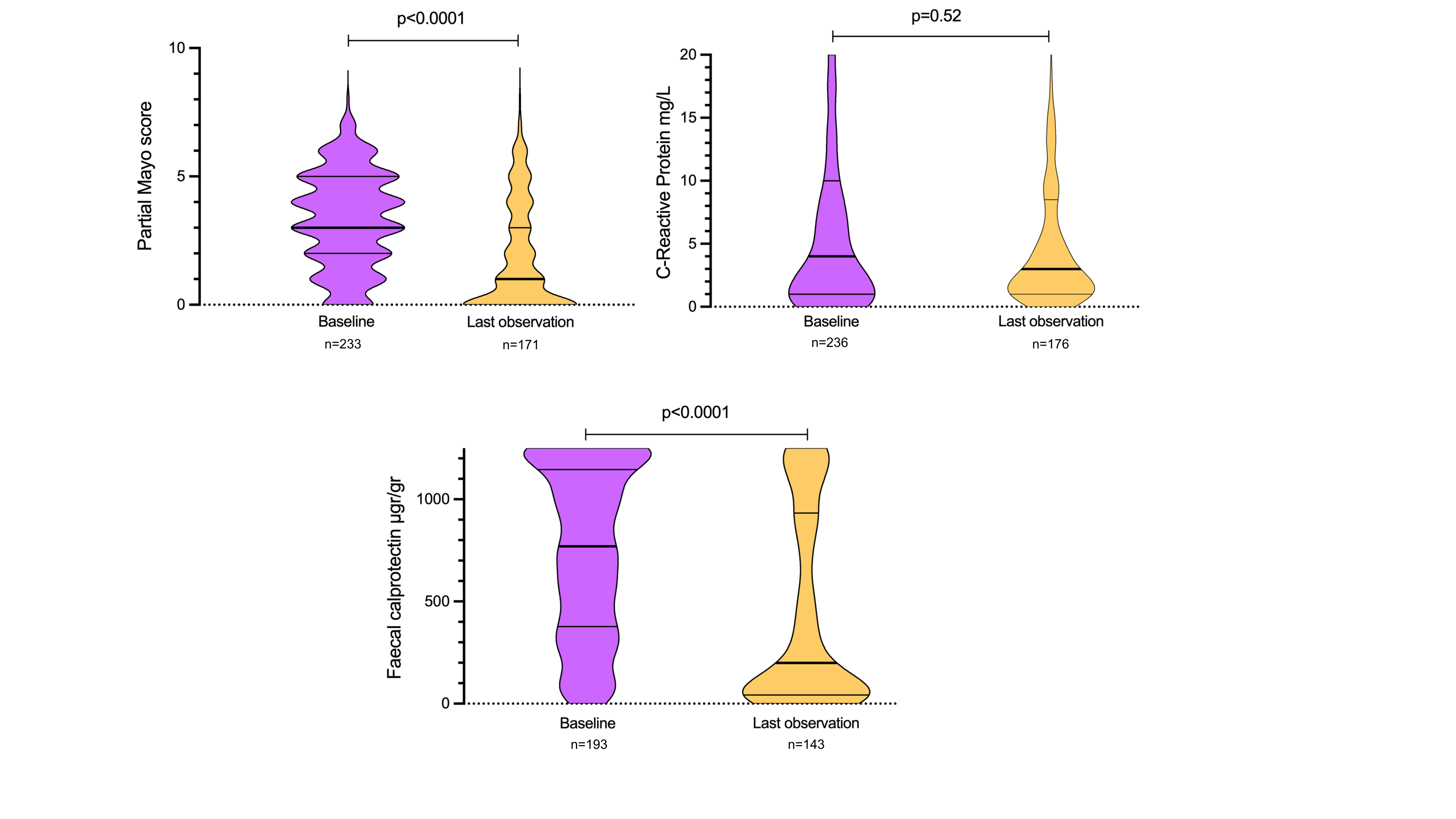
**

Baseline parameters compared to last clinical observation for the overall cohort; A) Partial Mayo score comparison. B) C-Reactive Protein; C) Faecal Calprotectin.

Mann–Whitney U test was used to compare each baseline median compared to last observation.

**FIGURE 2.**

**
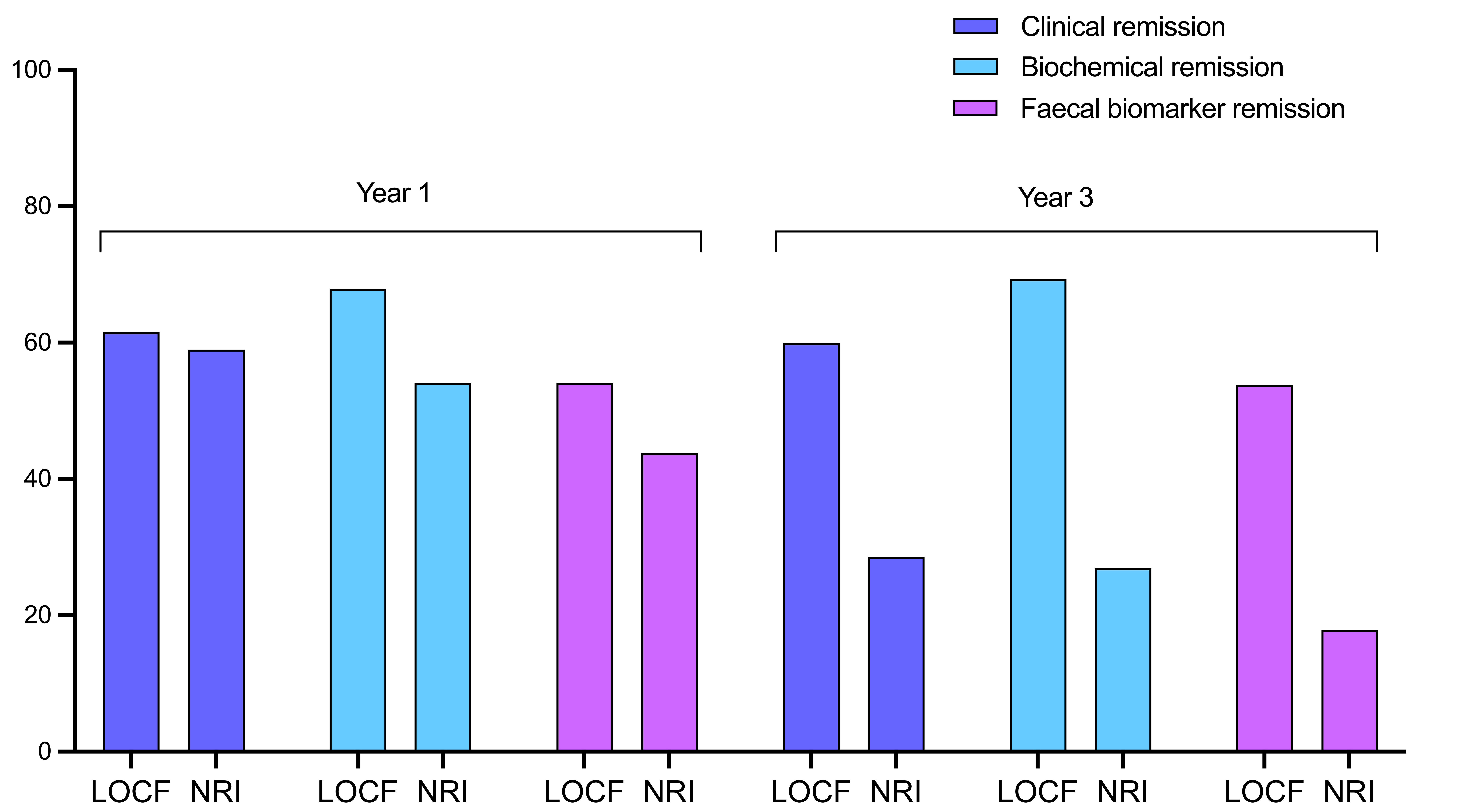
**

Clinical, biochemical and faecal biomarker remission at year 1 and year 3 using LOCF compared to NRI. LOCF: Last Observation Carried Forward; NRI: Non-Responder Imputation.

LOCF and NRI analysis of clinical, biochemical and faecal biomarker remission at year 1 and 3.

**FIGURE 3.
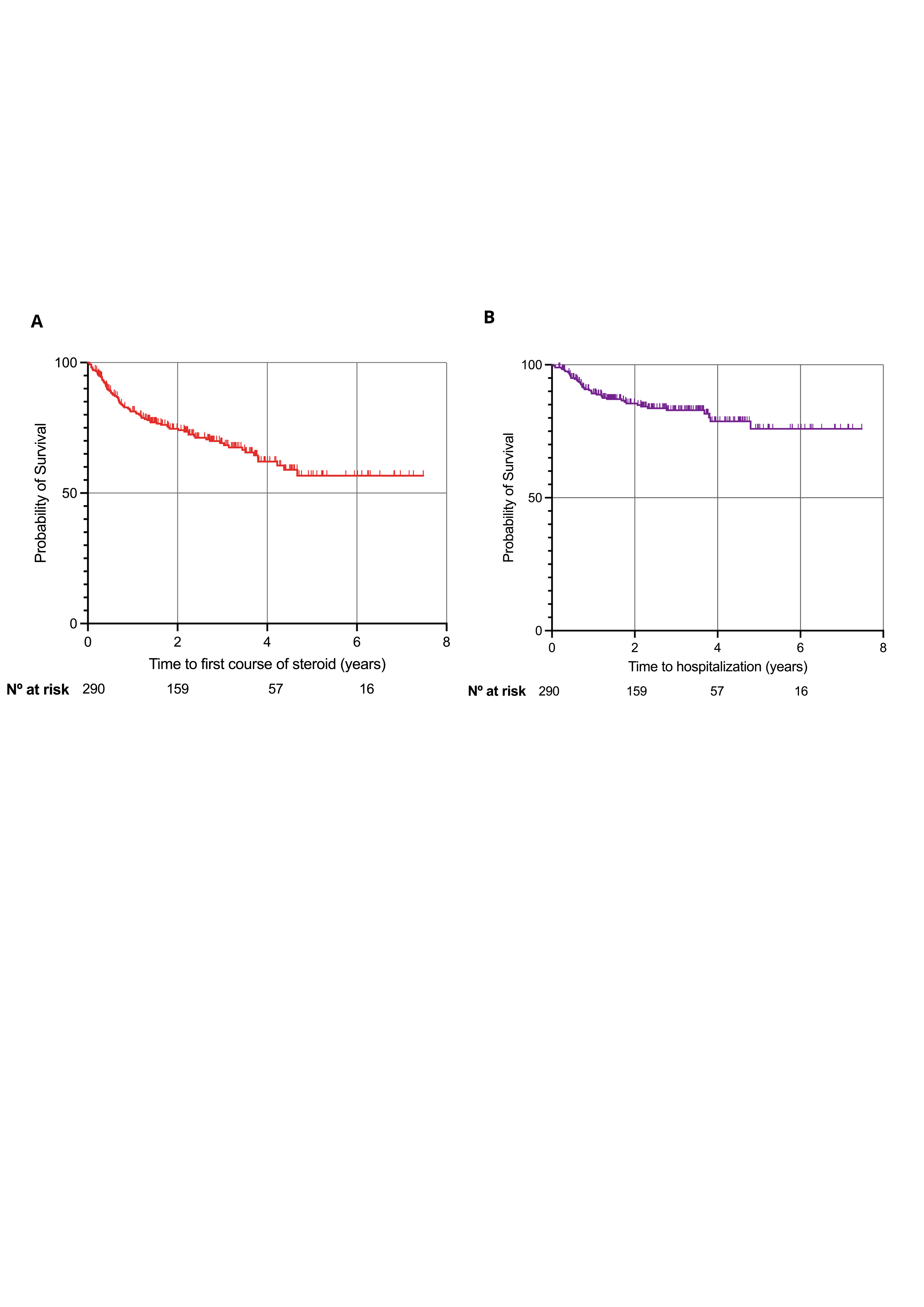
**

A) Kaplan-Meier curve of time to steroid prescription. B) Kaplan-Meier curve of time to hospitalization.
